# Prevalence of Human Parainfluenza Viruses and Noroviruses Genomes on Office Fomites

**DOI:** 10.1007/s12560-017-9327-z

**Published:** 2017-12-01

**Authors:** Agata Stobnicka, Małgorzata Gołofit-Szymczak, Angelina Wójcik-Fatla, Violetta Zając, Joanna Korczyńska-Smolec, Rafał L. Górny

**Affiliations:** 1 Central Institute for Labour Protection – National Research Institute, Czerniakowska 16 Street, 00-701 Warsaw, Poland; 2 Department of Health Biohazards and Parasitology, Institute of Rural Health, Jaczewskiego 2 Street, 20-090 Lublin, Poland; 3 Independent Public Healthcare Center, Wadowicka 3 Street, 34-122 Wieprz, Poland

**Keywords:** Parainfluenza, Norovirus, Office fomites, RT-qPCR

## Abstract

The aim of this study was to evaluate the potential role of office fomites in respiratory (human parainfluenza virus 1—HPIV1, human parainfluenza virus 3—HPIV3) and enteric (norovirus GI—NoV GI, norovirus GII—NoV GII) viruses transmission by assessing the occurrence of these viruses on surfaces in office buildings. Between 2016 and 2017, a total of 130 surfaces from open-space and non-open-space rooms in office buildings located in one city were evaluated for HPIV1, HPIV3, NoV GI, and NoV GII viral RNA presence. Detection of viruses was performed by RT-qPCR method. Study revealed 27 positive samples, among them 59.3% were HPIV3-positive, 25.9% HPIV1-positive, and 14.8% NoV GII-positive. All tested surfaces were NoV GI-negative. Statistical analysis of obtained data showed that the surfaces of office equipment including computer keyboards and mice, telephones, and desktops were significantly more contaminated with respiratory viruses than the surfaces of building equipment elements such as door handles, light switches, or ventilation tracts (*χ* ^2^ *p* = 0.006; Fisher’s Exact *p* = 0.004). All examined surfaces were significantly more contaminated with HPIVs than NoVs (*χ* ^2^ *p* = 0.002; Fisher’s Exact *p* = 0.003). Office fomites in open-space rooms were more often contaminated with HPIVs than with NoVs (*χ* ^2^ *p* = 0.016; Fisher’s Exact *p* = 0.013). The highest average concentration of HPIVs RNA copies was observed on telephones (1.66 × 10^2^ copies/100 cm^2^), while NoVs on the light switches (1.40 × 10^2^ copies/100 cm^2^). However, the Kruskal–Wallis test did not show statistically significant differences in concentration levels of viral RNA copies on surfaces between the all tested samples. This study unequivocally showed that individuals in office environment may have contact with both respiratory and enteric viral particles present on frequently touched surfaces.

## Introduction

According to World Health Organization, there are about 1.7 million deaths from diarrheal infections and about 1.5 million deaths from respiratory infections worldwide every year (Pruss-Ustun and Covalan 2006). As estimated, 60% of human diseases are induced by viral agents, and among them the most common illnesses are caused by respiratory and enteric viruses (Barker et al. 2001; McElhaney 2003).

From the medical point of view, human parainfluenza viruses (HPIVs), belonging to *Paramyxoviridae* family, are very important respiratory pathogens. There are four recognized serotypes of HPIVs: HPIV1, HPIV2, HPIV3, and HPIV4. HPIV1 and HPIV3 are the major causes of lower respiratory infections in children, the immunocompromised and chronically ill adults as well as among elderly (Collins et al. 1996; Falsey et al. 1995; Glezen et al. 1984, 2000; Liu et al. 2013; Whimbey et al. 1997). Infections in adults are usually less severe; however, serious infections caused by HPIVs among elderly and individuals with immunodeficiencies have been reported (Glasgow et al. 1995; Hendley 1990; Jarvis et al. 1979; Wendt et al. 1992). Symptoms of HPIV infections include cough, pharyngeal discomfort, nasal obstruction, coryza, sneeze, dyspnea, hoarseness, fever, chills, dizziness, headache, myalgia, and debilitation. Pathogenic activity of HPIVs in gastrointestinal illnesses is also suggested (Liu et al. 2013). According to Liu et al. (2013), HPIV1 and HPIV3 occurrences show seasonal trend with peak concentration on autumn turned to winter (September–November).

Noroviruses (Norwalk, NoV), belonging to *Caliciviridae* family, are the most common cause of epidemic gastroenteritis, responsible for at least 50% of all gastroenteritis outbreaks worldwide (Hall et al. 2011, 2013). Noroviruses have been divided into six established genogroups (GI-GVI) (Green 2013); however, provisional genogroup VII is also proposed (Vinjé 2015). NoV GII has emerged as the predominant NoV genotype in outbreaks of gastroenteritis worldwide (Huhti et al. 2011). Noroviruses are the most common pathogens associated with gastroenteritis in all age groups (Koopmans and Duizer 2004), however the most severe disease occurs in young children, among elderly, and persons with chronic illness (Estes et al. 2006). The infection is usually manifested by vomiting, diarrhea, nausea, fever, abdominal pain, headache, myalgia, general malaise, and chills. Norovirus-associated illnesses appeared to have winter-spring seasonality (January–March) (Arias et al. 2009; Marshall et al. 2005).

Viruses are probably the most common cause of infectious diseases, which are acquired indoors (Aitken and Jefferies 2001; Barker et al. 2001). Crowded indoor environments, like business office buildings, represent greater risk for dissemination of viral infections (La Rosa et al. 2013). The efficiency of transmission of viruses from hands to surfaces of often-touched objects depends on various factors such as species of the virus, the frequency and accuracy of hand-washing, and the physicochemical properties of specific surface (Julian et al. 2010). Available data suggest that viruses present on hands may contaminate the surface of 5–14 subsequently touched objects (Barker et al. 2004; von Rheinbaben et al. 2000). Viral contamination of one or two of the most touched surfaces in the office may result in the spread of viruses to 40–60% of the remaining surfaces within 2–4 h (Gerba 2014). At the room temperature, on non-porous surfaces, viruses keep their stability and infectious capacity (depending on environmental conditions) from 2 h to 1 week for HPIVs and from 3 to 4 weeks for NoVs, which suggest their important role in dissemination of infections among occupants (Doultree et al. 1999; Hendrickson 2003).

There are some evidences that contaminated surfaces may play a key role in the spreading of viral diseases (Boone and Gerba, 2007; Cheesbrough et al. 2000; Tuladhar et al. 2012), but knowledge concerning the role of specific surfaces and everyday-use-objects in viral transmission is still scarce. Infection control requires a clear understanding of viral transmission through everyday-use objects (Goldman 2000). Hence, the aim of this study was to evaluate the prevalence of parainfluenza viruses (HPIV1, HPIV3) and noroviruses (NoV GI, NoV GII) on frequently touched surfaces in office buildings during season conducive to respiratory and enteric infections.

## Materials and Methods

### Samples

Swabs from selected fomites were collected in office buildings located in Warsaw (Poland). A total of 130 samples, 65 from open-space (OS; ≥ 5 occupants) and 65 from non-open-space (NOS; < 5 occupants) office rooms, were evaluated over 9-month period (September 2016–May 2017). Samples were collected during regular work hours. Occupants were asked to leave, while the fomites were swabbed. Swabs for each virus were taken from 120 non-porous surfaces of everyday-use-objects (20 computer keyboards, 24 desktops, 22 computer mice, 24 phones, 18 handles, and 12 light switches) and ten ventilation ducts or air supply diffusers. Numbers of swab samples taken from studied objects were the same in OS and NOS office rooms. The samples were obtained from each surface using a sterile polyester fiber-tipped swabs prewetted in 0.9% saline solution, which ensures the most effective recovery of viruses from non-porous fomites (Ganime et al. 2015; Julian et al. 2011; Park et al. 2017). After swabbing, the samples were transported to the laboratory vertically in a special container at temperature of 4 °C. RNA isolation was performed within 24 h from collection of the swabs, which guarantees the highest efficiency of the isolation process.

### Viral RNA Extraction

RNA from swab samples was extracted with Viral Mini Kit PLUS (Syngen, Poland) based on silica column chromatography according to the procedure recommended by the manufacturer. An initial volume of 150 µl was used in the RNA extraction process to produce a final volume of 50 µl. Obtained RNA samples were stored at −80 °C until final analyze.

### Reverse-Transcription Quantitative PCR for Virus Detection

One-step reverse transcription quantitative polymerase chain reaction (RT-qPCR) was performed on a CFX96 Real-Time PCR thermocycler (BioRad, USA). Detection of HPIV1, HPIV3, NoV GI, and NoV GII was carried out using commercial kits, i.e., Human Parainfluenza Virus Type 1 (HPIV1) Haemagglutinin-Neuraminidase Glycoprotein, Human Parainfluenza Virus Type 3 (HPIV3) Haemagglutinin-Neuraminidase and Norovirus Genogroups 1 and 2—Norovirus GI capsid protein gene and Norovirus GII RNA dependent RNA polymerase gene (genesig^®^ PrimerDesign, Ltd., United Kingdom) dedicated to in vitro quantification of virus genomes. According to the manufacturer information, the applied kits were designed to have the possible broadest detection profile whilst remaining specific to the virus genomes. Based on a comprehensive bioinformatics analysis, the primers and probe sequences in these kits have 100% homology with a broad range of HPIV1, HPIV3, NoV GI, and Nov GII sequences, respectively.

Each reaction mixture (20 µl) contained 10 µl oasig^™^ lyophilised OneStep qRT-PCR MasterMix (PrimerDesign, Ltd.), 1 µl primer/probe mix, 1 µl internal extraction control primer/probe mix, 3 µl RNse-free water, and 5 µl of RNA sample. Reverse transcription was carried out at 55 °C for 10 min, followed by denaturation at 95 °C for 10 s. cDNA was immediately amplified with 49 cycles at 95 °C (2 min), 60 °C (60 s), and 60 °C (60 s). According to the manufacturer’s instruction, fluorogenic data were collected during the FAM and VIC channel. Negative and positive controls were included in each run. HPIVs’ and NoVs’ positive controls were purchased from PrimerDesign Ltd.

To minimize the potential contamination, all analytical steps including: RNA isolation, reagent preparation, sample preparation, and amplification were carried out in separate rooms. In all performed analyses, the sterile filter pipette tips free from RNase and DNase were only used.

RT-qPCR data were collected after the reaction and the threshold values defined the quantification cycle (Cq) were calculated by the BioRad CFX96 Manager Software. Samples with Cq ≤ 40 and with 25 ≤ Cq ≤ 31 for internal extraction control were considered as positive according to manufacturer’s instruction. Samples negative or with Cq > 40 were retested after 10-fold dilution, which allowed to evaluate possible presence of inhibitors (Fusco et al. 2017).

Quantification analysis was carried out with standard curves, constructed by amplification of tenfold dilutions of the positive control (standard from 1 to 1 × 10^6^ copies/reaction). Log RNA copies were plotted against Cq value. For each standard curve, amplification efficiency (*E*) was calculated as described by Fusco et al. (2017). The results expressed as the number of RNA copies per 100 cm^2^ of tested surfaces (copies/100 cm^2^) were calculated taking into account the volumes of solutions used for extraction and RNA isolation according to the following formula:

$$N_{{ 100{\text{cm}}^{ 2} }} = \, \left[ {\left( {N_{0} \times V_{ 2} /V_{ 1} } \right)/A} \right] \, \times { 1}00,$$

where $N_{{ 100{\text{cm}}^{ 2} }}$—number of RNA copies per 100 cm^2^, *N*_0_—starting quantity or RNA copies, *V*_2_—volume of solution used for extraction (µl), *V*_1_—volume of sample taken for isolation (µl), *A*—tested surface (cm^2^).

### Statistical Analysis

Statistical analyses were carried out with Kruskal–Wallis, Mann–Whitney U, *χ* ^2^, and Fisher Exact tests using STATISTICA, version 7.1 (StatSoft, USA). *p* values below 0.05 were considered statistically significant.

## Results

### Virus Detection by RT-qPCR

RT-qPCR-based studies showed the presence of HPIVs and NoVs on the examined surfaces. Overall, 20.8% of the samples were positive. Among positive samples, 59.3% of them were HPIV3-positive, 25.9% HPIV1-positive, and 14.8% NoV GII-positive. NoV GI was not detected on examined surfaces. The most commonly detected virus was HPIV3, followed by HPIV1 and NoV GII. HPIV3 dominated on office equipment objects such as computer keyboards (20% of the tested samples were positive), computer mice (18%), telephones (17%), and desktops (17%). HPIV1 was the most commonly detected on computer mice (9%), telephones (8%), and desktops (8%). In contrast to the HPIV3, HPIV1 was reported less frequently on the surfaces of computer keyboards (5%). NoV GII was detected on the light switches (17%) and door handles (11%). There was no viral RNA detected in the swab samples collected from ventilation system (Table 1).

**Table 1:** Number and percentage of virus-positive swab samples as identified by RT-qPCR

Virus detected	Number and percentage of positive samples (%)							Total number of analyzed samples
	Computer keyboard	Computer mouse	Telephone	Desktop	Door handle	Light switch	Ventilation system	
HPIV1	1/20 (5)	2/22 (9)	2/24 (8)	2/24 (8)	N.D.	N.D.	N.D.	7/130 (5)
HPIV3	4/20 (20)	4/22 (18)	4/24 (17)	4/24 (17)	N.D.	N.D.	N.D.	16/130 (12)
NoV GI	N.D.	N.D.	N.D.	N.D.	N.D.	N.D.	N.D.	N.D.
NoV GII	N.D.	N.D.	N.D.	N.D.	2/18 (11)	2/12 (17)	N.D.	4/130 (3)

*N.D* not detected

Taking into account the occurrence of co-contamination of tested surfaces, viral RNA of HPIV1 and HPIV3 were detected in the same time in case of two telephones, one computer keyboard, one computer mouse, and one desktop. Co-contaminated surfaces represent 3.8% of the total tested samples. There were no samples contaminated with NoV and HPIV in the same time.

Statistical analysis showed that the surfaces of office equipment (computer keyboards and mice, telephones, desktops) were significantly more contaminated with HPIVs than the surfaces of building components (door handles, light switches, ventilation system) (*χ* ^2^ *p* = 0.006; Fisher Exact test *p* = 0.004). All examined surfaces were significantly more contaminated with HPIVs than with NoVs (*χ* ^2^ test *p* = 0.002; Fisher Exact test *p* = 0.003).

Majority of HPIVs-positive samples came from OS rooms (74%), whereas NoVs-positive samples from NOS rooms (75%) (Table 2); however statistical analysis confirmed such prevalence of contamination for HPIVs only (*χ* ^2^ test *p* = 0.016; Fisher Exact test *p* = 0.013). The quantity of HPIVs-positive surfaces amongst the studied object categories varied between 66.7 and 83.3% and between 16.7 and 33.3% for OS and NOS rooms, respectively. In OS rooms, HPIVs were most often detected on computer mice (83.3%), while in NOS rooms on telephones and desktops (in both cases: 33.3%). In OS rooms, NoVs were only detected on door handles. Enteric viruses on light switches were found only in NOS rooms; however, all these samples (100%) were contaminated with them.

**Table 2:** Percentage of HPIV- and NoV-positive samples on fomites in open-space (OS) and non-open-space (NOS) rooms

Surface	Total number of positive samples	Percentage of positive samples (%)			
		Virus			
		HPIVs (HPIV1, HPIV3)		NoV GII	
		OS	NOS	OS	NOS
Keyboard	5	4/5 (80.0)	1/5 (20.0)	N.D.	N.D.
Computer mouse	6	5/6 (83.3)	1/6 (16.7)	N.D.	N.D.
Telephone	6	4/6 (66.7)	2/6 (33.3)	N.D.	N.D.
Desktop	6	4/6 (66.7)	2/6 (33.3)	N.D.	N.D.
Door handle	2	N.D.	N.D.	1/2 (50)	1/2 (50)
Light switch	2	N.D.	N.D.	N.D.	2/2 (100)
Total	27	74	26	25	75

*N.D.* not detected

### Standard Curves and Virus Quantification

For each virus, a standard curve was plotted based on the average Cq values of three replicates against the amount of copies/reaction. The efficiencies of amplification (*E* = 10^−1/S^–1, where *S* is the slope of linear regression curve) for each standard curve were as follows: *E*_HPIV1_ = 1.05 (*S* = − 3.20, *r* ^2^ = 0.98), *E*_HPIV3_ = 0.95 (*S* = – 3.45, *r* ^2^ = 0.99), *E*_NoV GI_ = 0.97 (*S* = – 3.56, *r* ^2^ = 0.98), and *E*_NoV GII_ = 1.04 (*S* = –3.24, *r* ^2^ = 0.99). The estimated detection level for tested viruses was 10 copies/reaction.

The mean quantities for tested viruses ranged between 10^1^ and 10^3^ RNA copies/100 cm^2^ (Table 3). The highest concentration of HPIV RNA copies was observed on telephones (average 1.66 × 10^3^ copies/100 cm^2^, range 4.89 × 10^2^ to 2.63 × 10^3^ copies/100 cm^2^). The average concentration of HPIV RNA copies on other surfaces were 3.36 × 10^2^, 4.85 × 10^2^, and 5.90 × 10^1^ copies/100 cm^2^ for computer keyboards, desktops, and computer mice, respectively. The highest concentration of NoV RNA copies was observed on light switches (average: 1.40 × 10^2^ copies/100 cm^2^, range: 1.10–1.70 × 10^2^ copies/100 cm^2^). The concentration of NoV RNA copies on door handles was detected at level of 5.06 × 10^1^ copies/100 cm^2^. However, the Kruskal–Wallis test did not show statistically significant differences in concentrations of RNA copies between the tested surfaces.

**Table 3:** Average concentrations of HPIVs and NoVs RNA copies/100 cm^2^ in positive swab samples (mean values and standard deviations, SD) regarding to object category

Surface	Virus			
	HPIVs (HPIV1, HPIV3)		NoV GII	
	Mean value	SD	Mean value	SD
Keyboard	3.36 × 10^2^	2.37 × 10^2^	N.D.	N.D.
Computer mouse	5.90 × 10^1^	2.20 × 10^1^	N.D.	N.D.
Telephone	1.66 × 10^3^	1.09 × 10^3^	N.D.	N.D.
Desktop	4.85 × 10^2^	4.65 × 10^2^	N.D.	N.D.
Door handle	N.D.	N.D.	5.06 × 10^1^	3.97 × 10^1^
Light switch	N.D.	N.D.	1.40 × 10^2^	8.09 × 10^1^

*N.D*. not detected

The average concentration of HPIV RNA copies was higher on surfaces located in OS rooms than in NOS ones (8.20 × 10^2^ vs. 2.89 × 10^2^ RNA copies/100 cm^2^) (Table 4). In case of NoV RNA copies, the opposite relation was observed (5.61 × 10^1^ vs. 8.70 × 10^1^ RNA copies/100 cm^2^); however, in both cases, the observed differences were not statistically significant (Mann–Whitney *U* test: *p* > 0.05).

**Table 4:** Average concentrations of HPIVs and NoVs RNA copies/100 cm^2^ in positive swab samples (mean values and standard deviations, SD) regarding to the type of room

Room	Virus			
	HPIVs (HPIV1, HPIV3)		NoV GII	
	Mean value	SD	Mean value	SD
OS	8.20 × 10^2^	9.47 × 10^2^	5.61 × 10^1^	2.83 × 10^0^
NOS	2.89 × 10^2^	1.63 × 10^2^	8.70 × 10^1^	5.13 × 10^1^

*OS* open-space rooms, *NOS* non-open-space rooms

## Discussion

There have been many studies examining the presence of bacteria and fungi on indoor fomites, but there is still lack of knowledge about viral nucleic acids, which can be found on everyday-use objects. Our study is the first investigation qualitatively and quantitatively analyzing presence of the most common respiratory and enteric viruses on everyday-use objects in the offices. Viral RNA of HPIVs and NoVs was detected on both office equipment (computer keyboards and mice, telephones, desktops) and building elements (door handles, light switches). This fact reinforces the theory that viruses can be easily spread within indoor environments (Goldman 2000). Viruses are probably the most common cause of infections spreading in buildings with large concentration of people (Brady et al. 1990). Many studies have documented the possibility of transferring the viruses from hands to the surfaces of touched objects and back, which may play an important role in spreading of viral infections (Ansari et al. 1988, 1991; Julian et al. 2010; Zhao et al. 2005).

In the current study, we found that both HPIV1 and HPIV3 viruses may be present on computer keyboards, mice, telephones, and desktops. It is consistent with Boone and Gerba (2010) observations describing the prevalence of HPIV1 on office equipment. However, in opposite to these authors, we did not find these viruses on door handles and light switches. The majority of HPIV-positive samples collected from office equipment seem to result from the transfer of contaminated nasal and respiratory secretions to fomites by touching (hand–nose/mouth-surface) and during coughing, sneezing, and talking. According to Couch et al. (1974), up to 10^7^ infectious virions can be present in 1 ml of respiratory secretions. When sneezing, they can travel up to 3 m at a speed of 20 m/s contaminating surrounding surfaces (Zhao et al. 2005).

NoVs were found only on door handles and light switches. These results confirmed the possibility of enteric virus occurrence on frequently touched objects, as described by Cheesbrough et al. (2000). Noroviruses may be numerously present in the saliva of infected individuals, but the highest concentrations of virions are observed in the feces and vomits (Atmar et al. 2008). The presence of NoVs on frequently touched surfaces suggests that they were transferred by contaminated hands. As indicated by Barker et al. (2004), NoVs virions present on the fingers may contaminate up to seven consecutive surfaces. These viruses can easily attach to stainless steel (e.g., door handles) or plastic (e.g., light switches) surfaces and survive on them from 4 to almost 6 days (Girard et al. 2010; Kim et al. 2014). In the case of enteric virus infection, a single vomiting incident can cause the release up to 30 million viral particles in the form of bioaerosol. As it was also showed, the virions released during the toilet flushing may survive in the air long enough to contaminate the surfaces of objects in immediate surroundings (Ansari et al. 1988; Zhao et al. 2005). Hence, it is worth mentioning that viral particles emissions to the environment can occur not only during illness but also before the symptoms appear as well as for a few days or weeks after the disease has ended (Goldman 2000). In case, when hygiene level is insufficient, a transfer of the virus from hands to the touched surfaces is very possible.

In the present study, swab samples from non-porous surfaces were analyzed. According to Abad et al. (1994), viruses usually survive for longer time on non-porous surfaces compared to porous ones. The stability of viruses on surfaces may vary and depend on several factors. In indoor environments, both UV radiation and pH of the surfaces have a limited influence, whereas temperature and relative humidity can play an important role in viral genome stability; however, simultaneous impact of these factors is difficult to assess (Memarzadeh 2012). According to Choi et al. (2014), viral single-stranded RNA is less resistant to degradation by high temperature than double-stranded DNA.

Viral genome stability and infection ability may increase or decrease depending on the number of microbes present on the surfaces (Boone and Gerba 2007). Bacteria and fungi may exhibit protective effects against virions (securing against drying and disinfection). On the other hand, the effects of bacterial proteases and fungal enzymes can adversely influence the environmental stability of viruses (Boone and Gerba 2007; Sobsey and Meschke 2003).

Considering viral infectious capacity, specific infectious dose is difficult to determine. According to the Infectious Disease Epidemiology Section at the Department of Health and Hospitals in Louisiana, NoVs infectious dose is estimated as 10 viral particles introduced into the body per os (Louisiana Office of Public Health 2013). Other data indicate that less than 10 virions are sufficient to infect a healthy adult (Hutson et al. 2004). Similar information is provided by the Public Health Agency of Canada (PHAC). Hall (2012) determined the norovirus infectious dose at the level of ≥ 18 viral particles. In turn, animal studies show that infectious *per os* dose of human NoVs GII for gnotobiotic pigs range from 10^3^ to 10^4^ viral RNA copies (Bui et al. 2013). According to PHAC, HPIVs infectious dose is unknown; however, some authors indicate that for HPIV1, ≥ 1.5 units of virus is enough to induce infection when viruses are introduced into the body through the nasal drops (Collins and Kennedy 1999). Taking into account all these information, the results obtained in the current study from office buildings showed that viruses present on tested surfaces may play important role in infections of occupants, especially in situation when personal hygiene is insufficient (e.g., when there are no hand washing before leaving the toilet, using single-use handkerchiefs, hand washing after sneezing or coughing etc.). On the other hand, it should be clearly stated that the positive samples obtained in this study may contain both infectious and non-infectious viral particles as detected with RT-q PCR method. Although one-step RT-qPCR enables both qualitative and quantitative analyses of viral RNA, this method does not allow assessing viral infectious ability (Rodríguez et al. 2009). A big step forward in the determination of viral infectivity is the viability PCR (vPCR). In this method, through the use of a simple sample pre-treatment with specific intercalating reagents (e.g., viability dyes like propidium monoazide or ethidium monoazide), it is possible to distinguish viral RNA from non-intact viral particles (Topping et al. 2009). On the other hand, an attempt to use viability dyes for the detection of infectious noro- or influenza viruses with RT-qPCR was not so far successful (Parshionikar et al. 2010; Graiver et al. 2010; Elizaquível et al. 2013). Hence, the assessment of infectivity of the viruses transferred from fomites to humans needs further investigations.

The surfaces of office objects like telephones, computer keyboards, mice or desktops, as well as door handles and light switches are frequently used but rarely cleaned and disinfected. HPIVs can be efficiently removed from the surfaces with a majority of commonly used detergents, disinfectants or antiseptic agents (Brady et al. 1990). However in case of NoVs, the outbreak investigations indicated that enteric viruses are relatively resistant to common disinfectants (Cheesbrough et al. 2000; D’Souza et al. 2006). According to Girard et al. (2010), only sodium hypochlorite was sufficiently effective against NoVs, while alcohols and ammonium-based disinfectants were less efficient.

Based on this study, it can be concluded that individuals in office environment may have contact with viral RNA present on different frequently touched surfaces. Keeping the personal hygiene on a proper level as well as regular and effective cleaning of the surfaces (including desktops, computer keyboards and mice, telephones, handles, light switches etc.) with proper disinfectants degrading viral RNA should reduce the possibility of virus transmission and infections among occupants.
